# Unexpected diversity in the host-generalist oribatid mite *Paraleius leontonychus* (Oribatida, Scheloribatidae) phoretic on Palearctic bark beetles

**DOI:** 10.7717/peerj.9710

**Published:** 2020-09-11

**Authors:** Sylvia Schäffer, Stephan Koblmüller

**Affiliations:** Institute of Biology, University of Graz, Graz, Austria

**Keywords:** Scolytinae, Oribatid mite, Genetic structure, Species delimitation, Cryptic species

## Abstract

Bark beetles are feared as pests in forestry but they also support a large number of other taxa that exploit the beetles and their galleries. Among arthropods, mites are the largest taxon associated with bark beetles. Many of these mites are phoretic and often involved in complex interactions with the beetles and other organisms. Within the oribatid mite family Scheloribatidae, only two of the three nominal species of *Paraleius* have been frequently found in galleries of bark beetles and on the beetles themselves. One of the species, *P. leontonychus*, has a wide distribution range spanning over three ecozones of the world and is believed to be a host generalist, reported from numerous bark beetle and tree species. In the present study, phylogenetic analyses of one mitochondrial and two nuclear genes identified six well supported, fairly divergent clades within *P*. *leontonychus* which we consider to represent distinct species based on molecular species delimitation methods and largely congruent clustering in mitochondrial and nuclear gene trees. These species do not tend to be strictly host specific and might occur syntopically. Moreover, mito-nuclear discordance indicates a case of past hybridization/introgression among distinct *Paraleius* species, the first case of interspecific hybridization reported in mites other than ticks.

## Introduction

Bark beetles (Scolytinae) are a species-rich subfamily of weevils (Curculionidae), that play important ecological roles in structuring natural plant communities and large-scale biomes (Raffa, Gregoire & Lindgren, 2015). Only a few outbreaking species are responsible for the poor public image of bark beetles, as they can kill healthy trees by mass-attacks inflicting serious damage to forestry and thus cause extensive ecological and economic losses, also with regard to protected forests etc. In general, however, bark beetles are important nutrient recyclers and consequently important components of and likely beneficiary to these ecosystems (Cobb et al., 2010).

Bark beetles also support a large number of other taxa (e.g., nematode, fungi, bacteria) that exploit the beetles and their galleries and have either a positive or a negative impact on the fitness of the beetles (Moser, 1975; Six, 2012). Among arthropods, mites are the largest taxon associated with bark beetles. More than 100 mite species have been reported as potential associates so far. These mites are typically phoretic and often involved in complex interactions, for example, beetle-mite-fungus, suggesting that the mites’ phoresy on beetles is not just a means of transport (Vitzthum, 1926; Hofstetter, Moser & Blomquist, 2014). In most of these mites, the second nymphal stage (deutonymph) and adult females are phoretic. The close phoretic relationship is often accompanied by special morphological adaptations enabling mites to attach to the host; for example, phoretic uropodine deutonymhs can form an anal pedicel, phoretic scutacarid females have enlarged tarsal claws on leg I and many astigmatine hypopi possess a complex sucker plate posterior to legs IV (Krantz, 2009). Among the many mite species known to be associated with bark beetles, 17 belong to the species-rich (~10.000 described species) order Oribatida (Hofstetter, Dinkins-Bookwalter & Klepzig, 2015). Very little is known about potential interactions between Oribatida and bark beetles, that is, the role of these mites in the communities in the beetles’ galleries. Although some oribatids have been repeatedly found in these galleries or pheromone traps (Moser & Bogenschuütz, 1984; Norton, 1980; Ahadiyat & Akrami, 2015), it is still unclear whether they should be regarded as part of a real subcortical merocoenosis, or just migrate—for example, in search for food—from the outer bark, which they usually inhabit, into the galleries (Kielczewski, Moser & Wisniewski, 1981). Within the oribatid family Scheloribatidae only one genus, *Paraleius* Travé, 1960, has been frequently found in galleries of bark beetles and on the beetles themselves (Moser & Bogenschuütz, 1984; Pernek et al., 2012; Knee, 2017).

The genus *Paraleius* comprises three nominal species, *Paraleius leahae* Knee, 2017, *Paraleius strenzkei* (Travé, 1960) and *Paraleius leontonychus* (Berlese, 1910). All three can be easily identified based on diagnostic morphological characters. *Paraleius leahae* occurs in Canada (Knee, 2017), *P. strenzkei* in France (Pyrenees; Weigmann (2006)) and *P. leontonychus* is widespread with records in three ecozones of the world, Nearctic, Neotropics and Palearctic (Ahadiyat & Akrami, 2015). Two of these species, *P. leahae* and *P. leontonychus* are known to disperse phoretically on scolytine species. As morphological adaptation, both phoretic species exhibit a strong hook-like claw on each tarsus, which allows them to adhere to the host (Vitzthum, 1926; Knee, 2017). Whereas *P. leahae* has been found to be associated with *Hylastes porculus* Erickson,1836 and *Dendroctonus valens* LeConte, 1860 (Knee, 2017), *P. leontonychus* has been collected from at least 25 bark beetle species from various host trees (Ahadiyat & Akrami, 2015). In Europe, *P. leontonychus* has been found associated with 14 beetle species. Unfortunately, older literature contains only little to no information on both bark beetle species and respective host tree. Nonetheless, the most frequent records of European *P. leontonychus* are with *Ips typographus* (Linnaeus, 1758) found on Norway spruce (*Picea abies* (L.) Karst.) or in pheromone traps (Moser & Bogenschuütz, 1984; Moser, Eidmann & Regnander, 1989; Penttinen, Viiri & Moser, 2013). Moreover, it has been found in connection with bark beetle infestations on silver fir (*Abies alba* Mill.; Pernek et al., 2008, 2012) or the maritime pine (*Pinus pinaster* Alton, 1789; Fernández, Diez & Moraza, 2013).

Recent phylogenetic studies on mites and metabarcoding studies based on environmental DNA suggest that the number of mite species currently recognized is a considerable underestimation of the true diversity, even for common taxa allegedly easy to identify to species level (Navia et al., 2013; Young et al., 2019; Schäffer, Kerschbaumer & Koblmüller, 2019). As *P. leontonychus* has been reported to be associated with a large number of bark beetles, which themselves tend to show some preferences to particular tree genera (Raffa, Gregoire & Lindgren, 2015), the question arises whether *P. leontonychus* is a truly widespread species with no host preference at all or whether it is a complex of cryptic species with specialization to particular hosts and trees as has been previously documented for uropodoid mites phoretic on bark beetles (Knee et al., 2012).

Against this background, we used DNA sequences of the second half of the mitochondrial COI region (COI-2) and two nuclear markers, the D3 region of the 28S rRNA (D3-28S) and the 18S rRNA (18S), to (i) assess the diversity and genetic structure of *P. leontonychus* originating from different bark beetle-infested tree species and, (ii) test whether *P. leontonychus* is indeed a host generalist, or rather a complex of several (cryptic) species with potential host specificity.

## Materials and Methods

### Sampling

**Figure 1:**
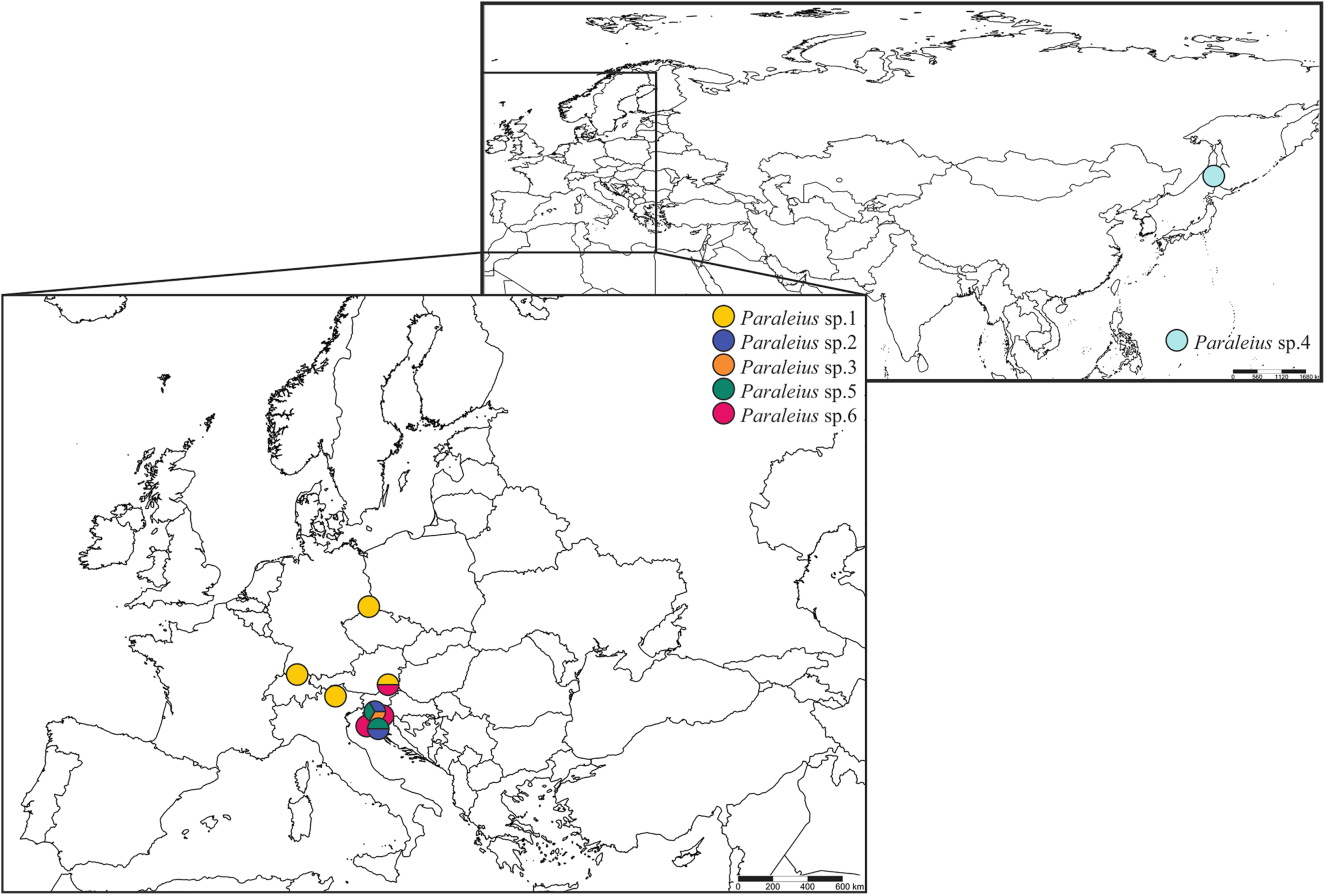
Maps with sampling sites of *Paraleius* species for the present study.

Bark of four tree species infested with different bark beetles and ethanol-preserved mite specimens were collected between 2015 and 2017. Some samples were provided by local colleagues: Alexandr A. Khaustov (Russia), Milan Pernek (Croatia) and Peter Stephen (South Africa). Moreover, Heinrich Schatz provided verbal permission for the collection of Italian samples and the Provincial Government of Styria (department 13; GZ: ABT13-53W-50/2018-2) for all Styrian samples (Austria). Mite specimens were extracted from these bark samples with Berlese-Tullgren-Funnels and immediately preserved in absolute ethanol. Two individuals were found in propylene glycol-preserved sampling material from pheromone traps in Germany (sampling: Peter Wilde) and Switzerland (sampling: Joël Loop), attracting the European spruce bark beetle (*I. typographus*). We chose representatives of two closely related genera, *Dometorina plantivaga* (Berlese, 1895) (Dp) and *Siculobata sicula* (Berlese, 1892) (Ss), as outgroup taxa. Species were identified based on character traits given in Weigmann (2006) and Knee (2017). Detailed information and photographs on the included *Paraleius* individuals and their associated bark beetle species found in the specific tree sample are given in Table 1, Fig. 1 and Fig. S1. Maps were created in SimpleMappr online service (Shorthouse, 2010).

**Table 1:** Information on sampling site, substrate, GenBank accession numbers of investigated genes and derived mitochondrial (mt) group for each *Paraleius* specimen in the present study.

Sample ID	Location	Country	Coordinates		Substrate	COI-2	D3-28S	18S	mt Group
R8_10_2	Puch	AUT	46.931075	15.764967	Norway spruce	MT462281	MT396663	MT406359	G1
R8_20_2	Puch	AUT	46.931075	15.764967	Norway spruce	MT462282	MT396664	MT406360	G1
R36_1	Delnice	HRV	45.420000	14.747217	Silver fir	MT462275	MT396657	MT406353	G6
R36_2	Delnice	HRV	45.420000	14.747217	Silver fir	MT462276	MT396658	MT406354	G6
R45_1	Paldau	AUT	46.939982	15.777055	Norway spruce	MT462277	MT396659	MT406355	G1
R45_3	Paldau	AUT	46.939982	15.777055	Norway spruce	MT462278	MT396660	MT406356	G6
R45_4	Paldau	AUT	46.939982	15.777055	Norway spruce	MT462279	MT396661	MT406357	G6
R57_2	Oberstorcha	AUT	46.962122	15.790404	Norway spruce	MT462280	MT396662	MT406358	G6
Pop09B_06	Litorić	HRV	45.412936	15.077517	Silver fir	MT462256	MT396638	MT406334	G2
Pop09B_08	Litorić	HRV	45.412936	15.077517	Silver fir	MT462257	MT396639	MT406335	G2
Pop09B_14	Litorić	HRV	45.412936	15.077517	Silver fir	MT462258	MT396640	MT406336	G5
Pop09B_21	Litorić	HRV	45.412936	15.077517	Silver fir	MT462259	MT396641	MT406337	G3
Pop09B_28	Litorić	HRV	45.412936	15.077517	Silver fir	MT462260	MT396642	MT406338	G2
Pop10B_03	Krk	HRV	45.020253	14.571320	Aleppo pine	MT462261	MT396643	MT406339	G5
Pop10B_05	Krk	HRV	45.020253	14.571320	Aleppo pine	MT462262	MT396644	MT406340	G5
Pop10B_13	Krk	HRV	45.020253	14.571320	Aleppo pine	MT462263	MT396645	MT406341	G2
Pop32F_12	Friedersdorf	DEU	51.03279	14.575399	Trap	MT462264	MT396646	MT406342	G1
Pop39B_05	Pula	HRV	44.852277	13.831892	Aleppo pine	MT462265	MT396647	MT406343	G6
Pop39B_14	Pula	HRV	44.852277	13.831892	Aleppo pine	MT462266	MT396648	MT406344	G6
Pop39B_15	Pula	HRV	44.852277	13.831892	Aleppo pine	MT462267	MT396649	MT406345	G6
Pop46B_13	Karersee	ITA	46.409973	11.577228	Norway spruce	MT462268	MT396650	MT406346	G1
Pop46B_32	Karersee	ITA	46.409973	11.577228	Norway spruce	MT462269	MT396651	MT406347	G1
Pop55B_01	Sakhalinskaya Oblast	RUS	46.784150	142.385500	Sakhalin spruce	MT462270	MT396652	MT406348	G4
Pop55B_02	Sakhalinskaya Oblast	RUS	46.784150	142.385500	Sakhalin spruce	MT462271	MT396653	MT406349	G4
Pop55B_03	Sakhalinskaya Oblast	RUS	46.784150	142.385500	Sakhalin spruce	MT462272	MT396654	MT406350	G4
Pop55B_04	Sakhalinskaya Oblast	RUS	46.784150	142.385500	Sakhalin spruce	MT462273	MT396655	MT406351	G4
Pop62F_16	Flaach, Buch am Irchel	CHE	47.540467	8.612300	Trap	MT462274	MT396656	MT406352	G1
SsBl01	Pietermaritzburg	ZAF	−29.526278	30.505167	Lemon tree	MT462283	MT396665	MT406361	outgroup
SsBl02	Pietermaritzburg	ZAF	−29.526278	30.505167	Lemon tree	MT462284	MT396666	MT406362	outgroup
SsBl05	Pietermaritzburg	ZAF	−29.526278	30.505167	Lemon tree	MT462285	MT396667	MT406363	outgroup
DpGU1	Hart bei Graz	AUT	47.063022	15.505042	Apple tree	MT462254	MT396636	MT406332	outgroup
DpGU2	Hart bei Graz	AUT	47.063022	15.505042	Apple tree	MT462255	MT396637	MT406333	outgroup

### DNA extraction, PCR amplification and sequencing

Single individuals were used for the extraction of total genomic DNA applying the Chelex protocol given in Schäffer et al. (2018). PCR amplification and sequencing followed standard protocols (Schäffer et al., 2010, 2018). We obtained sequences of a 622 bp fragment of COI-2, a 314-320 bp fragment of D3-28S and a 1,748–1,784 bp fragment of the 18S gene (primer pairs are same as in references before). All sequences were edited in MEGA 6.0 (Tamura et al., 2013) and verified by comparisons with known oribatid mite sequences from GenBank. All sequences are available from GenBank with the accession numbers given in Table 1.

### Phylogenetic analyses

Sequences of COI-2 were aligned by eye in MEGA, those of the two nuclear markers were aligned using the MAFFT v.7 web version (Katoh, Rozewicki & Yamada, 2017). The D3-28S and 18S alignments had a final length of 320 and 1,784 bp, respectively. All alignments are available in the Supplemental Material. Mean uncorrected pairwise distances (*p*-distances) of pre-defined groups were calculated in MEGA. For visualization of the barcoding gap in the three markers, we used the package “spider” (Brown et al., 2012) in R (R Core Team, 2019). Introgressed individuals from Pop39 were excluded (for details see “Results”).

To select the best-fitting model of molecular evolution for each gene, the Akaike Information Criterion (AIC) in the “Smart Model Selection” (SMS; Lefort, Longueville & Gascuel, 2017) implemented in the PhyML 3.0 online execution (Guindon et al., 2010; http://www.atgc-montpellier.fr/phyml/) was applied. Phylogenetic inference of single gene datasets was based on Maximum Likelihood (ML) and Bayesian inference (BI), conducted in PhyML and MrBayes 3.2.4 (Ronquist et al., 2012), respectively, implementing the models selected by SMS. ML analyses were performed under default parameter settings and nodal support was assessed by means of bootstrapping (1,000 replicates). The Bayesian Markov Chain Monte Carlo simulations were conducted in two independent runs with four chains, each for 10 (COI-2)/15 (D3-28S, 18S) million generations sampled every 100th (18S)/1,000th (COI-2, D3-28S) generation. All parameter values were initially assessed graphically in Tracer v1.7 (Rambaut et al., 2018; available at http://beast.community/tracer) and different burn-ins were applied to allow likelihood values to reach stationarity (standard deviation of split frequencies was <0.01).

PopArt (Leigh & Bryant, 2015) was used to infer haplotype networks based on statistical parsimony.

### Species delimitation analyses and species tree

#### Single-locus delimitation based on COI-2

Several methods were employed for species delimitation based on the COI-2 data. ABGD (Puillandre et al., 2012) was used via a graphic web interface (https://bioinfo.mnhn.fr/abi/), applying default parameters and uncorrected p-distances. Furthermore, two tree-based approaches, the “Generalized Mixed Yule Coalescent” (GMYC; Fujisawa & Barraclough, 2013) model implemented in the “splits” package (Ezard, Fujisawa & Barraclough, 2017) for R (R Core Team, 2019) and the “Poisson Tree Process” (PTP) model (Zhang et al., 2013; Kapli et al., 2017) were applied. ML partition PTP (PTP–ML) was run on the bPTP web-server (http://species.h-its.org/) using standard default settings and the Bayesian MCC tree as input. As GMYC and the Bayesian version of GMYC (bGMYC) require ultrametic tree(s) as input data, the respective guide tree(s) were obtained in BEAST2 (models and settings are given in Table S2). Convergence and stationarity of chains were again checked in Tracer. The first 30% of sampled trees were excluded as burn-in and a MCC tree was calculated from all remaining trees. Using this MCC tree, two GMYC analyses with (i) the single (sGMYC) and (ii) the multiple (mGMYC) thresholds setting were conducted. The benefit of the bGMYC approach is the use of many trees in order to account for uncertainty in tree space (Reid & Carstens, 2012). Therefore, we resampled the trees from the posterior distribution of the Beast runs at lower frequency in LogCombiner part of the BEAST v2.4.2 package (Bouckaert et al., 2014). The resulting data set finally comprised 344 trees which were used for bGMYC analysis conducted in R, using the bGMYC package (Reid & Carstens, 2012).

#### Multi-locus delimitation

As multi-locus approach we used the Bayesian species delimitation method BP&P v3.1 implemented in bppX v1.2.2 (Yang & Rannala, 2010; Yang, 2015), which analyzes multi-locus sequence data under the multispecies coalescent prior, accounting for gene tree conflicts due to incomplete lineage sorting (Yang & Rannala, 2010). All three markers were used as input data, running an unguided analysis (called “A11”), which simultaneously performs species delimitation and species tree inference (Yang & Rannala, 2010, 2014; Yang, 2015). Based on their clustering in the single gene BI trees, individuals were assigned into monophyletic groups. As bppX only merges different groups into one species, never trying to split them into multiple ones (Yang, 2015), we tested two different approaches: (UG1) following the results from the COI-2 analyses, individuals were assigned to eight clusters (G1-G6, Dp, Ss); (UG2) individual assignment was identical to UG1, with the sole difference that specimens of G6 were split into two groups, assuming a putative seventh *Paraleius* species (G6A included the five individuals R36_1&2, R45_3&4 and R57_2, G6B all three individuals from Pop39B). UG 2 would therefore allow for possible merging of groups/species, especially in the case of wrong a priori assignments. Since prior distributions on population size (θ) and species divergence time (τ) can have an impact on the posterior probabilities (PP) for models and no empirical data are available for the study species, we followed the procedure in Lin, Stur & Ekrem (2018), applying seven different combinations of priors: (i) θ ~ G(2,10), τ ~ G(2,20); (ii) θ ~ G(2,10), τ ~ G(2,2000), (iii) θ ~ G(2,10), τ ~ G(2,500), (iv) θ ~ G(2,100), τ ~ G(2,200); (v) θ ~ G(2,100), τ ~ G(2,2000); (vi) θ ~ G(2,1000), τ ~ G(2,200); (vii) θ ~ G(2,1000), τ ~ G(2,2000).

In addition, a multi-locus species tree based on all three genes was inferred using StarBEAST2 (Ogilvie, Bouckaert & Drummond, 2017; implemented in the BEAST2 package). Species assignment followed a conservative approach, excluding introgressed individuals from Pop39 (for details see “Results”). Models and settings are given in Table S2. Resulting log-files were evaluated in TRACER and all treefiles were combined using LogCombiner, after discarding an initial burn-in of 30% of trees and resampling every 100th tree. DensiTree (Bouckaert, 2010) was used to visualize the species tree.

## Results

**Figure 2:**
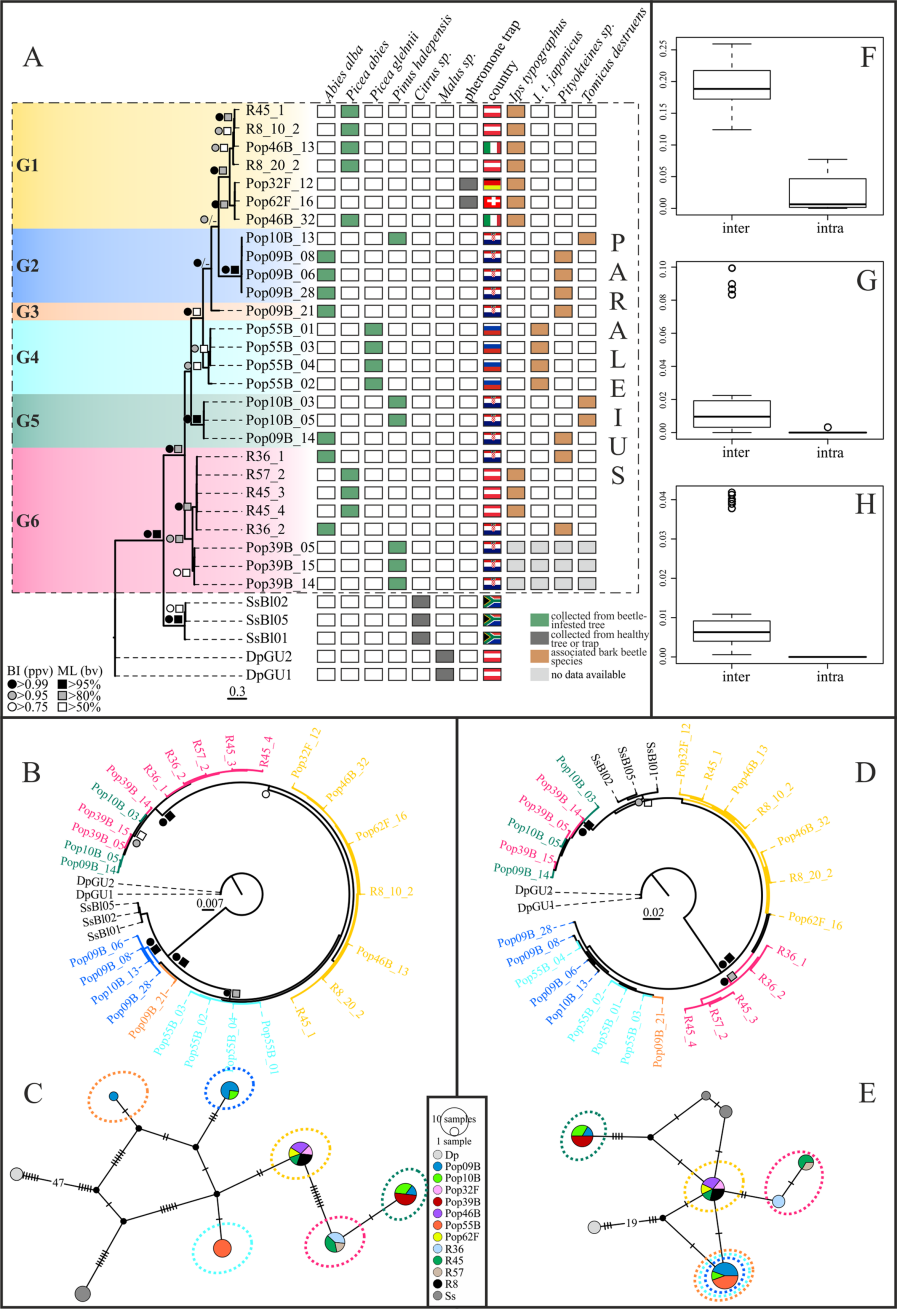
Bayesian inference trees based on COI-2 (A), 18S (B) and D3-28S (D) gene of the *P. leontonychus* species complex. Additionally, 18S (C) and D3-28S (E) haplotype networks are provided. (F–H) Intra-and interspecific distances (uncorrected *p*-distances). Symbols at nodes represent Bayesian posterior probability values (ppv) and bootstrap values (bv) for ML. Individual sampling information (host beetle, country, tree species) is presented as matrix next to the COI-2 BI tree. Different colors correspond to the six putative species.

Maximum Likelihood and BI tree searches yielded highly similar tree topologies. Therefore, only the BI trees are presented here (Figs. 2A, 2B and 2D). Phylogenetic inference based on the COI-2 gene indicated, with good to high statistical support, six distinct *P. leontonychus* clusters (G1-6), of which G3 was only represented by a single individual (Fig. 2A). Though the exact branching order among the main clusters was different, similar results were obtained from the 18S data, except for three individuals originating from Pula (Pop39B), placed in cluster G6 based on the CO-2 data, that grouped with all specimens from cluster G5 (Fig. 2B). All remaining G6 specimens from localities R36, R45 and R57 grouped together. In contrast, the D3-28S dataset yielded a poorly resolved phylogeny, supporting only some of the six mitochondrial *Paraleius* groups (Fig. 2D). Regarding the mitochondrial groups G5 and G6, however, D3-28S revealed the same result as the 18S dataset with the clustering of individuals from Pula (Pop39B) in cluster G5, which here resulted as fairly divergent from cluster G6 (Fig. 2D).

Uncorrected *p*-distances between the six mitochondrial *Paraleius* groups ranged from 12.7% to 19.6% in the COI, from 0.1% to 0.7% in the 18S and from 0% to 2.2% in the D3-28S gene. Within-group distances ranged from 0% to 4.7% in the COI gene and from 0% to 0.3% in the two nuclear genes (see Table S3). Thus, a distinct barcoding gap was only evident in COI-2 (Figs. 2F–2H).

COI-2 haplotype diversity was very high in the present dataset, for example, out of the 27 *Paraleius* specimens examined, a total of 20 haplotypes were identified. Groups G2, G3 and G5 consisted solely of specimens with Croatian origin, and G5 contained individuals from Russia. On three single tree trunks we found more than one *Paraleius* group, namely two in samples R45 and Pop10B, and three in Pop09B (Figs. 2A, 2C and 2E).

**Figure 3:**
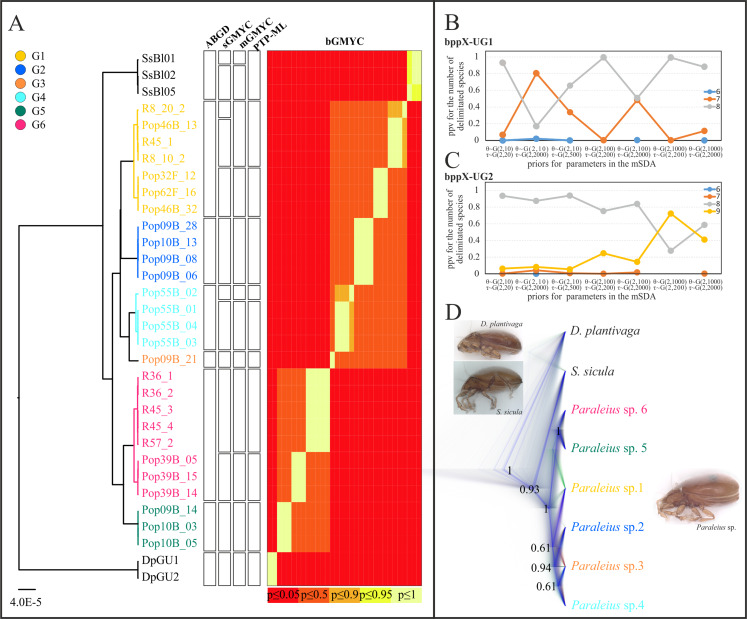
Species delimitation analyses (A) based on single and (B–D) multi locus data. (A) Ultrametric COI-2 tree and results of five different SDAs are given. (B and C) BP&P graphs show the posterior probabilities for the number of putative species under different sets of priors. (D) Species tree of six *Paraleius* species presented as cloudogram. The darker the lines the more likely the respective branch. Only posterior probabilities >0.5 are shown. Colors of clades are same as in Fig. 1. Photo credit: M. Kerschbaumer.

Based on COI-2, single-locus delimitation based on COI-2 (sSDA) inferred 8–13 putative species, including outgroups: ABGD yielded 8, PTP-ML 11 and all other methods 12 or 13 putative species (Fig. 3A). In all analyses, G2, G3, G5 and both outgroup species (except for mGMYC which split *S. sicula* in two clusters) formed separate clusters. In all but one (ABGD) of the five sSDAs, G1, G4 and G6 were split into two, respectively, three clusters. For G1, mGMYC and PTP-ML suggested two clusters, while sGMYC indicated three. The apparent over-splitting of these groups is probably caused by a small overall sample size and comparatively large within-group distances, for example, up to 4.7% in G1 or 2.4% in G4 (Table S3).

Results of both multi-locus delimitation (mSDAs) favored the existence of 8 putative species in our dataset (depending on the prior used with moderate to high support; Figs. 3B and 3C), matching the result from the ABGD analysis. All UG1 and UG2 analyses, under different prior settings, delimited Dp, Ss and *Paraleius* G1-4 as separate species with high statistical support (PP= 0.96–1.0, Table 2). With regards to groups G5 and G6, most UG1 analyses delimited them as two separate species, however, runs under the priors small θ and deep τ suggested G5 and G6 rather as one than two species (Table 2). All seven UG2 analyses recovered G6A as a single species (PP= 0.99997–1.0) and favored a one-species scheme for G5 and G6B. Under the priors small population size and large divergence time (θ ~ G(2, 1,000), τ ~ G(2, 200)), however, bppX delimited nine species with moderate support for G5 and G6B being separate species (Table 2).

**Table 2:** Delimited species and their posterior probabilities of the BP&P species delimitation analysis using different sets of priors for ancestral population size (θ) and root age (τ). Two different approaches of individual assignments were tested (UG1, UG2).

Prior distributions	Delimited species and their posterior probabilities—bppX-UG1									
	*Paraleius*								*S. sicula*	*D. plantivaga*
	G1	G2	G3	G4	G5	G6	G5 & G6			
θ ~ G(2, 10), τ ~ G(2, 20)	1.0	0.99991	0.99790	0.99799	0.93345	0.93345	0.06655		0.99999	0.99999
θ ~ G(2, 10), τ ~ G(2, 2,000)	0.99999	0.99212	0.97757	0.98159	0.17725	0.17725	0.82275		0.99855	0.99372
θ ~ G(2, 10), τ ~ G(2, 500)	1.0	0.99919	0.99632	0.99701	0.66184	0.66184	0.33816		0.99987	0.99939
θ ~ G(2, 100), τ ~ G(2, 200)	1.0	1.0	0.99999	0.99999	0.99688	0.99688	0.00312		1.0	1.0
θ ~ G(2, 100), τ ~ G(2, 2,000)	1.0	0.99790	0.98825	0.99121	0.51555	0.51554	0.48445		0.99958	0.99875
θ ~ G(2, 1000), τ ~ G(2, 200)	1.0	0.99998	0.99995	0.99997	0.99619	0.99619	0.00381		1.0	1.0
θ ~ G(2, 1,000), τ ~ G(2, 2,000)	1.0	0.99925	0.99606	0.99693	0.88828	0.88828	0.11172		0.99993	0.99987
	**Delimited species and their posterior probabilities—bppX-UG2**									
	***Paraleius***								***S. sicula***	***D. plantivaga***
	**G1**	**G2**	**G3**	**G4**	**G5**	**G6A**	**G6B**	**G5 & G6B**		
θ ~ G(2, 10), τ ~ G(2, 20)	1.0	0.99973	0.99780	0.99801	0.06440	1.0	0.06440	0.93560	1.0	1.0
θ ~ G(2, 10), τ ~ G(2, 2,000)	0.99999	0.98658	0.96112	0.96636	0.08659	0.99992	0.08659	0.91338	0.99848	0.99342
θ ~ G(2, 10), τ ~ G(2, 500)	1.0	0.99891	0.99395	0.99519	0.07339	1.0	0.07339	0.92661	0.99983	0.99934
θ ~ G(2, 100), τ ~ G(2, 200)	1.0	0.99990	0.99924	0.99932	0.24308	1.0	0.24308	0.75692	1.0	1.0
θ ~ G(2, 100), τ ~ G(2, 2,000)	1.0	0.99712	0.98109	0.98485	0.14210	0.99997	0.14209	0.85787	0.99948	0.99833
θ ~ G(2, 1,000), τ ~ G(2, 200)	1.0	1.0	0.99997	0.99997	0.71971	1.0	0.71971	0.28029	1.0	1.0
θ ~ G(2, 1,000), τ ~ G(2, 2,000)	1.0	0.99900	0.99523	0.99640	0.41163	1.0	0.41163	0.58837	0.99993	0.99976

In the multi-locus species tree analysis of the six putative *Paraleius* species the exact branching order of some species was statistically only weakly supported (Fig. 3D). Only the sister group relationship of G5 and G6 was well supported with a PP = 1.0; a clade comprising G2, G3 and G4 received moderate support (PP = 0.94).

## Discussion

Here, we provide the first phylogenetic analysis of the allegedly widespread scheloribatid mite species, *P. leontonychus*. Based on phylogenetic analysis of one mitochondrial, two nuclear markers and Species delimitation analyses (SDAs) we show that *P. leontonychus* is not a single species, but rather a complex of several cryptic lineages. Specifically, our analyses suggested the existence of at least six distinct groups within *P. leontonychus* (G1-6). Some SDA algorithms split some of these groups even further. However, many SDA methods are affected by the geographical and taxonomic scope of the sampling and might be particularly sensitive to small intra-specific sample sizes (Lim, Balke & Meier, 2012). Given the limited overall sample size and low divergence within the six main groups, as compared to inter-group divergences, we regard these additional groups as artifacts. The fact that syntopically found specimens congruently clustered in the single gene trees derived from COI-2 and 18S sequence data (and also the D3-28S data, considering the poor resolution of this marker), additionally indicates that there is no ongoing gene flow between the respective groups. Genetic distances, based on the COI sequences, among these six main groups amounted to 12.7–19.6% (uncorrected *p*-distances), which is well in line with previously reported species-level divergences in other oribatid mites (Heethoff et al., 2007; Pfingstl, Baumann & Lienhard, 2019; Schäffer et al., 2010; Schäffer, Kerschbaumer & Koblmüller, 2019) and suggests that this radiation is not of recent origin. Despite intensive investigations, it was not possible to differentiate these groups morphologically. Using the original description by Berlese (1910) in this context is of no help, as it is very short and only general in scope (no details). Nonetheless, all herein investigated specimens correspond well to the previous re-descriptions and/or illustrations by various authors (Vitzthum, 1926; Travé, 1960; Wunderle, Beck & Woas, 1990, Mahunka, 1996; Weigmann, 2006; Ahadiyat & Akrami, 2015), showing no conspicuous differences. Contrary to *P. leahae* and *P. strenzkei*, our individuals possess hetero-tridactylous tarsi with one median, hook-like claw and two thin and fine lateral claws, a diagnostic character for *P. leontonychus*. The exterior of *Paraleius* is quite inconspicuous, consisting of a very limited set of character traits that might be useful for species delimitation. Moreover, there are no hints from literature (due to for example, morphological peculiarities) that *P. leontonychus* might be a complex of more than one species. Though, if there are morphological differences between the *Paraleius* groups these will be only minor. An additional method to differentiate the groups would be (geo-)morphometric analyses. In our case, however, this application is impossible, as all specimens were crushed at the beginning of the DNA extraction as we did not expect this high cryptic diversity, with distinct species occurring syntopically on single tree trunks (see Pop09B, Pop10B and R45). Apart from these morphological aspects, the genetic data clearly support the acceptance of groups G1-6 as true biological species. We, therefore, designate them as *Paraleius* species1-6 (=*Paraleius* sp1-6; see Fig. 3D) in the following.

The locus typicus for *P. leontonychus* is Filettino on Monte Viglio, east of Rome, Italy. As none of the herein studied individuals originates from or close to the locus typicus and since the original description of the species provides no details on host tree and beetle, it remains unclear, which of the six species represents the true *P. leontonychus* and whether the species was included at all in our dataset.

Assessing the taxonomic diversity in cryptic lineages with little or no ecological and/or morphological divergence is still one of the major challenges in systematic biology (Fišer, Robinson & Malard, 2018), but necessary, as informed estimates on the true diversity of taxa are an important prerequisite for our understanding of their evolutionary significance and role in ecosystems. Recent studies have shown that inconspicuous and/or small taxa, often with a reclusive life style, show particularly high levels of cryptic diversity (Von Saltzwedel, Scheu & Schaefer, 2017; Wagner et al., 2019). Mites are no exception, and indeed, their actual diversity seems to be vastly underestimated, even in common and easily recognizable taxa (Navia et al., 2013; Young et al., 2019; Schäffer, Kerschbaumer & Koblmüller, 2019).

Even though the branching order among the main *Paraleius* groups differed among the three gene trees (phylogenetic relationships were generally poorly resolved in the D3-28S tree) and thus received only moderate support in the species tree, the individual samples by and large resulted in the same main clusters in the mitochondrial and nuclear trees. The only exceptions were three individuals of mitochondrial G6 that grouped with the samples of mitochondrial G5 in the nuclear gene trees, a pattern generally suggestive for either introgression or incomplete lineage sorting (ILS). Distinguishing between these two potential causes of mito-nuclear discordance is not trivial. However, discordance as result of ILS is expected to have no predictable biogeographic patterns (Funk & Omland, 2003; Toews & Brelsford, 2012). As the incongruence concerns a single locality and since the phylogenetic placement of the questionable samples was concordant in the two nuclear gene trees, we assume that introgression, rather than ILS, is the underlying cause for the detected discordance. Among mites, hybridization has hitherto been only reported for ticks (Ixodidae) (Rees, Dioli & Kirkendall, 2003; Kovalev, Golovljova & Mukhacheva, 2016; Patterson et al., 2017) and our data thus provide the first indication that hybridization/introgression might occur also in other mite taxa. In theory, sex-biased dispersal can also cause mito-nuclear discordances. In *Paraleius*, however, contrary to most other phoretic mites, both males and females seem to be phoretic (Pernek et al., 2012; Knee, 2017). Hence, we do not consider sex-biased dispersal as potential cause of the observed mito-nuclear discordance.

*Paraleius leontonychus* is regarded as a broad host generalist, reported from quite a few scolytine beetle species (Knee, 2017). Most of the mite specimens included in the present study were found together with beetle species already known to be associated with *P. leontonychus*. Additionally, for the first time, we found *Paraleius* associated with *Tomicus destruens* (Wollaston, 1865) on Aleppo pine (*Pinus halepensis* Mill.).

Despite the limited sample size, our analyses indicate that *Paraleius* species do not tend to be strictly host specific, that is, associated with only a single bark beetle and/or tree species. With the exception of *P*. sp. 1, species for which more than one locality was sampled were found together with more than one beetle species, which themselves were, based on our limited sample size, strictly associated with particular tree species (Fig. 2A). In addition, single beetle and tree species are hosts for several *Paraleius* species. Thus, *P*. sp. 2, 3, 5 and 6 were found together with *Pityokteines* sp. on *A. alba* and *P*. sp. 2, 5 and 6 with *T. destruens*, and *P*. sp. 1 and 6 were found to be associated with *I. typographus* on *P. abies*. Four of the six *Paraleius* species were collected in Croatia, indicating that *Paraleius* diversity might be high in the Balkans, likely driven by complex patterns of range reductions and expansion of host beetle and tree species in the course of recurrent glacial cycles during the Quaternary (Horn et al., 2009; Litkowiec, Lewandowski & Rączka, 2016). However, we expect that species diversity is higher than previously assumed also in other refugial areas of the host trees, particularly in areas (forests) with several different host tree species.

Unlike the other European species, *P*. sp. 1 was not found in Croatia but only in Central Europe, possibly due to a strong preference for Norway spruce, which is the dominant forest tree in this region. Interestingly, *P*. sp. 2, which was collected in far eastern Russia on Sakhalin spruce (*Picea glehnii* (F. Schmidt) Mast.) together with *I. typographus f. japonicus* Niijima, 1909, neither constitutes a distinctly divergent lineage (if geography was the main driver of *Paraleius* diversification) nor the sister species of *P*. sp. 1 (if host beetle species relationships were the main determinant of their diversification), but rather represents one of six roughly equally divergent *Paraleius* species. In general, even if they might prefer certain tree species, most bark beetles are not specific to single host tree species, but do colonize a number of—mostly phylogenetically related—species (Bertheau et al., 2009). Considering the complex Quaternary population dynamics of both bark beetles and host trees, the relaxed host specificity of bark beetles and the large number of bark beetle species associated with *Paraleius*, single *Paraleius* species might get easily distributed across a range of tree species. As a consequence, previously allopatric *Paraleius* species may have been brought into sympatry, facilitating occasional hybridization/introgression.

## Conclusions

*Paraleius leontonychus* has been known as a widespread species associated with a large number of bark beetle hosts. We find, however, that *P. leontonychus* is not a single widespread host generalist mite species, but rather a complex of several morphologically cryptic species. These species are probably no strict host specialists and occur at least in part in sympatry (and even syntopy). Our data already point to a stunning diversity and call for large scale phylogeographic sampling, ideally combined with multi-locus or genomic data to fully resolve the complex evolutionary history and patterns of potential host specificity in this species complex. Although our study only covers a part of the total distribution range and the potential hosts of *P. leontonychus*, we already demonstrate that there are more than one *Paraleius* species phoretic on European bark beetles. To ensure that future studies in both basic and applied research are aware of this situation, it is very important to present these first insights on the unexpected genetic diversity of the allegedly widespread Palearctic *P. leontonychus*.

## Supplemental Information

10.7717/peerj.9710/supp-1 Supplemental Information 1 Photographs of representatives of the three studied genera. Arrows point to one generic main character trait, the sublamella, which is distinct in *Dometorina*, weak in *Siculobata* and absent in *Paraleius*. Photo credit: M. Kerschbaumer

10.7717/peerj.9710/supp-2 Supplemental Information 2 Best-fitting substitution models selected by the Akaike Information Criterion (AIC) in the “Smart Model Selection” (SMS) implemented in PhyML.

10.7717/peerj.9710/supp-3 Supplemental Information 3 Models and settings used in BEAST and StarBEAST2 analyses.

10.7717/peerj.9710/supp-4 Supplemental Information 4 Mean uncorrected pairwise distances (1) between and (2) within clades/species. (1) Values for COI-2 gene are given in gray boxes and for 18S (upper value), respectively, 28S-D3 (lower value) in white boxes. (2) n/c = not possible to estimate evolutionary distances because of insufficient sample size. G1-6 refer to the six detected mitochondrial *Paraleius* groups; *Dometorina* (*D*.) *plantivaga* and *Siculobata* (*S*.) *sicula* represent outgroup species. Red values indicate no interspecific genetic differences.

10.7717/peerj.9710/supp-5 Supplemental Information 5 Alignment of the 18S rRNA gene.

10.7717/peerj.9710/supp-6 Supplemental Information 6 Alignment of the D3 region of the 28S rRNA.

10.7717/peerj.9710/supp-7 Supplemental Information 7 Alignment of the COI-2 gene.

## Additional Information and Declarations

### Field Study Permissions

The following information was supplied relating to field study approvals (i.e., approving body and any reference numbers): Some samples were provided by local colleagues: Alexandr A. Khaustov (Russia), Milan Pernek (Croatia) and Peter Stephen (South Africa). Moreover, Heinrich Schatz provided verbal permission for the collection of Italian samples and the Provincial Government of Styria (department 13; GZ: ABT13-53W-50/2018-2) for all Styrian samples (Austria). Two included individuals were collected from pheromone traps in Germany (sampling: Peter Wilde) and Switzerland (sampling: Joël Loop).

### Data Availability

The following information was supplied regarding data availability: All alignments are available as a Supplemental File. All sequences are available from GenBank (Table 1).
