# Supplementary figures and images for: Unexpected diversity in the host-generalist oribatid mite Paraleius leontonychus (Oribatida, Scheloribatidae) phoretic on Palearctic bark beetles
Source: PeerJ. 2020 Sep 11;8:e9710. doi: 10.7717/peerj.9710 (PMC7489242; doi:10.7717/peerj.9710)

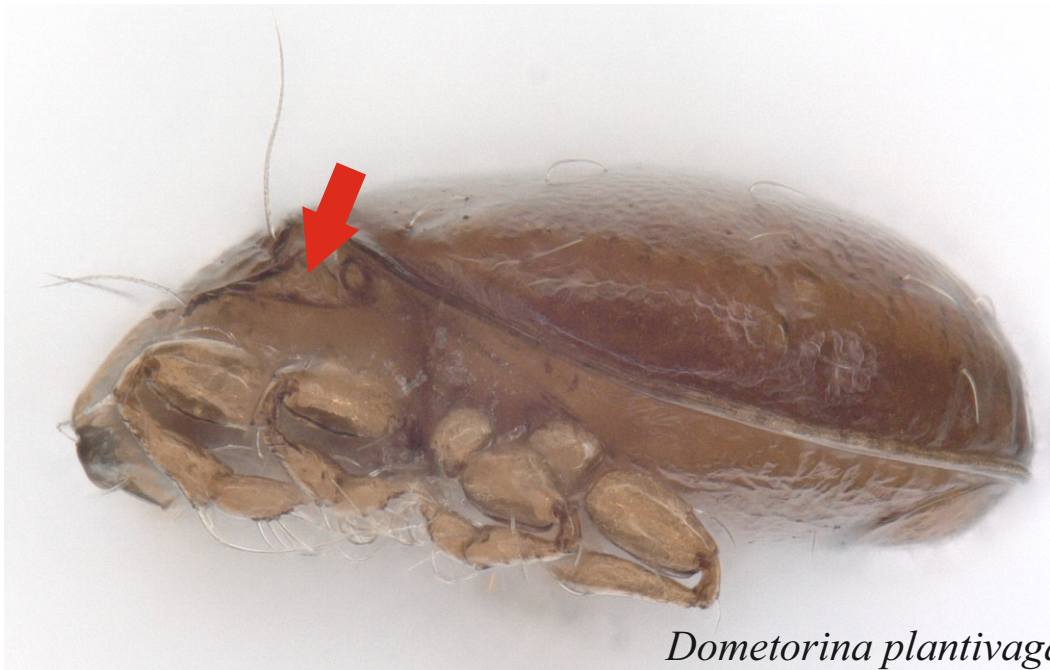

*Dometorina plantivaga*

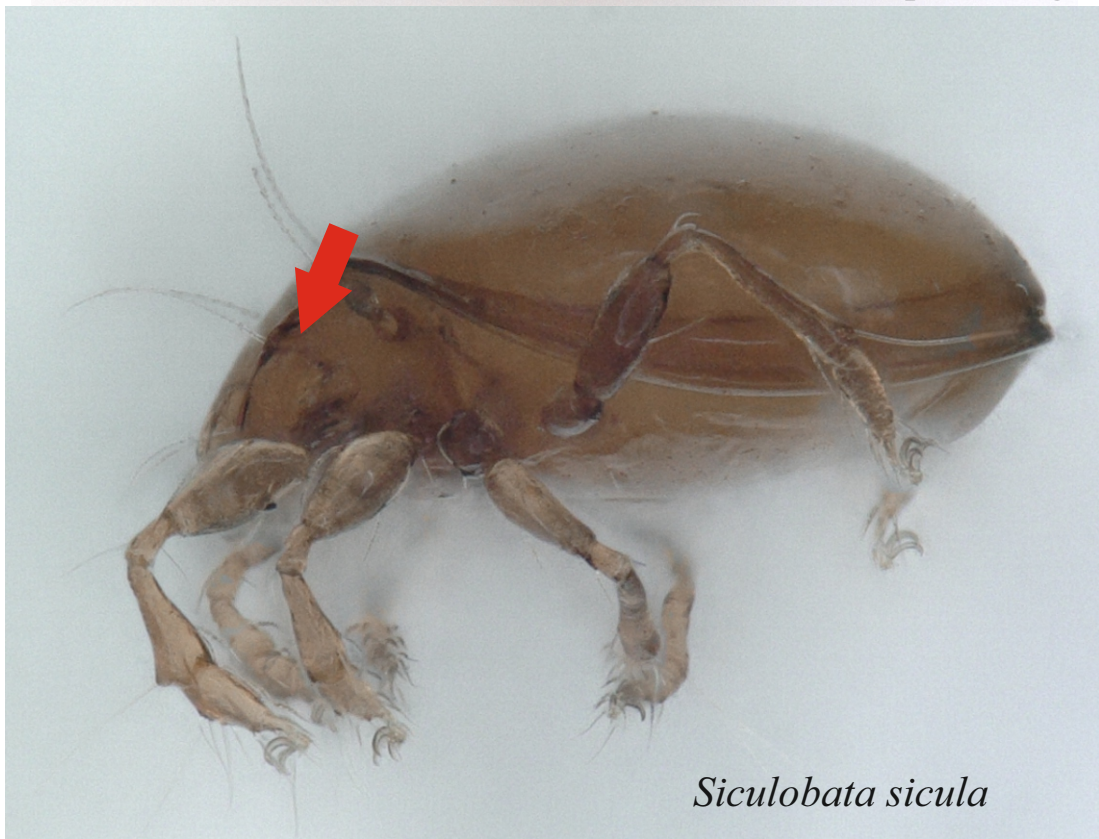

*Siculobata sicula*

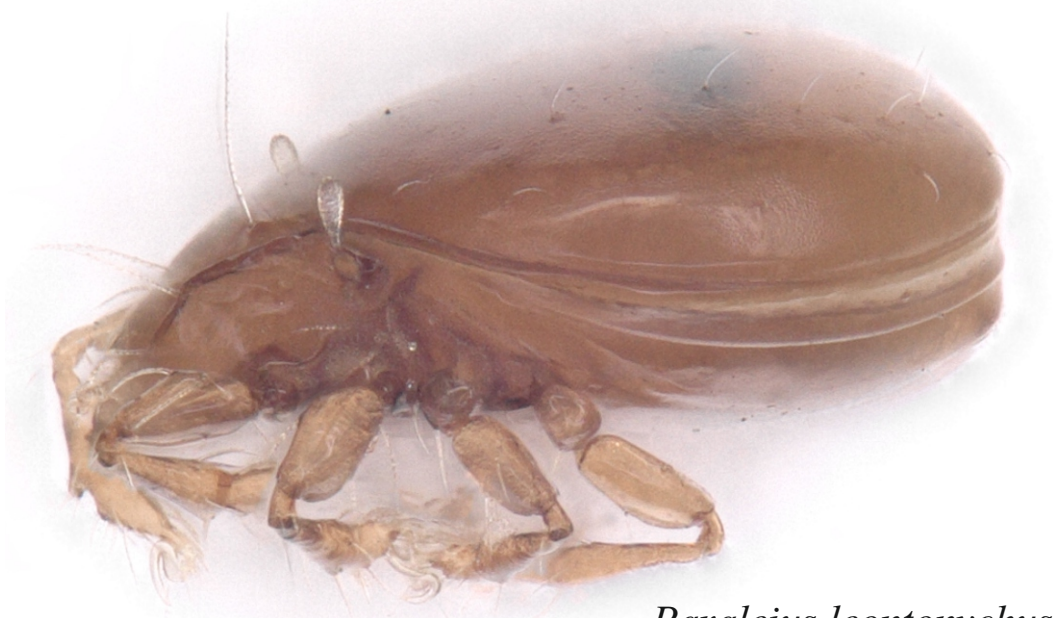

*Paraleius leontonychus*

Supplement: Supplemental Information 1 — Photo credit: M. Kerschbaumer [file peerj-08-9710-s001.pdf]
